# Histone methyltransferase Ash1L mediates activity-dependent repression of neurexin-1α

**DOI:** 10.1038/srep26597

**Published:** 2016-05-27

**Authors:** Τao Zhu, Chen Liang, Dongdong Li, Miaomiao Tian, Sanxiong Liu, Guanjun Gao, Ji-Song Guan

**Affiliations:** 1 MOE Key Laboratory of Protein Sciences, Tsinghua-Peking Center for Life Sciences, School of Life Sciences, Center for Brain-Inspired Computing Research, IDG/McGovern Institute for Brain Research, Tsinghua University, Beijing 100084, China

## Abstract

Activity-dependent transcription is critical for the regulation of long-term synaptic plasticity and plastic rewiring in the brain. Here, we report that the transcription of neurexin1α (nrxn1α), a presynaptic adhesion molecule for synaptic formation, is regulated by transient neuronal activation. We showed that 10 minutes of firing at 50 Hz in neurons repressed the expression of nrxn1α for 24 hours in a primary cortical neuron culture through a transcriptional repression mechanism. By performing a screening assay using a synthetic zinc finger protein (ZFP) to pull down the proteins enriched near the nrxn1α promoter region *in vivo*, we identified that Ash1L, a histone methyltransferase, is enriched in the nrxn1α promoter. Neuronal activity triggered binding of Ash1L to the promoter and enriched the histone marker H3K36me2 at the nrxn1α promoter region. Knockout of Ash1L in mice completely abolished the activity-dependent repression of nrxn1α. Taken together, our results reveal that a novel process of activity-dependent transcriptional repression exists in neurons and that Ash1L mediates the long-term repression of nrxn1α, thus implicating an important role for epigenetic modification in brain functioning.

Activity-dependent gene expression is closely associated with neuronal plasticity. Neuronal activity regulates the expression levels of many genes, including those for the transcriptional factors Egr1, Fos and Npas4 and the synaptic proteins GluR1, NMDAR1, BDNF^1^,^2^,^3^,^4^,^5^. Such activity-dependent gene expression is essential for the long-term regulation of neuronal functions and plasticity underlying the physiology and pathology of memory and addiction^6^,^7^,^8^. In addition to CREB-mediated transcriptional regulation^9^, epigenetic mechanisms have been identified as essential for the regulation of activity-dependent transcription^10^,^11^,^12^,^13^. Histone modifications, such as methylated H3K9, methylated H3K27 and methylated H4K20, are associated with silent, non-transcribed genes, whereas tri-methylation of H3K4 and acetylation of histones are correlated with transcriptional activation^14^. Some histone modifications have bidirectional functions in gene regulation. For example, methylated H3K36 has been implicated in both transcriptional activation and repression^15^,^16^,^17^. As a major problem for the epigenetic regulation of mediated transcriptional regulation, the specificity of such epigenetic regulation is hardly observed on the specific gene loci that regulate gene expression^18^. Until now, there have been no tools available to identify the unknown factors that regulate activity-dependent gene expression *in situ*.

Neurexins are cell adhesion molecules that are essential for synapse formation^19^,^20^. The mammalian genome contains three neurexin genes, including nrxn1, 2 and 3. Each of the genes encodes two major groups of transcripts, α- and β-neurexins, using two distinct promoters^21^,^22^,^23^. α- and β-neurexins share identical intracellular domains. Between them, α-neurexin has a larger extracellular domain. Mice lacking α-neurexins showed defects in vesicle exocytosis and a reduction in inhibitory synapses^24^,^25^. Consistent with the essential roles of neurexins in synapse formation and synaptic plasticity, the mutants of this gene have been widely observed in many neurological diseases, including schizophrenia, autism spectrum disorders (ASDs) and epilepsy^26^,^27^,^28^,^29^,^30^. However, the regulation mechanism underlying neurexin expression is still unclear.

Here, we report that the expression of nrxn1α in primary cortical neuron cultures underwent activity-dependent repression. To identify the mechanism for its transcriptional control, we developed an affinity-based method to purify the transcription regulators for the nrxn1α promoter directly from the brain. We found that Ash1L binds to the nrxn1α promoter in neurons. Neuronal activity recruits Ash1L to the nrxn1α promoter and enriches H3K36me2 at the promoter. By generating *Ash1L*-deficient mouse via CRISPR-cas9 mediated genome editing, we showed that Ash1L is essential for the activity-dependent repression of nrxn1α.

## Results

### Activity-dependent repression of neurexin-1α

**Figure 1:**
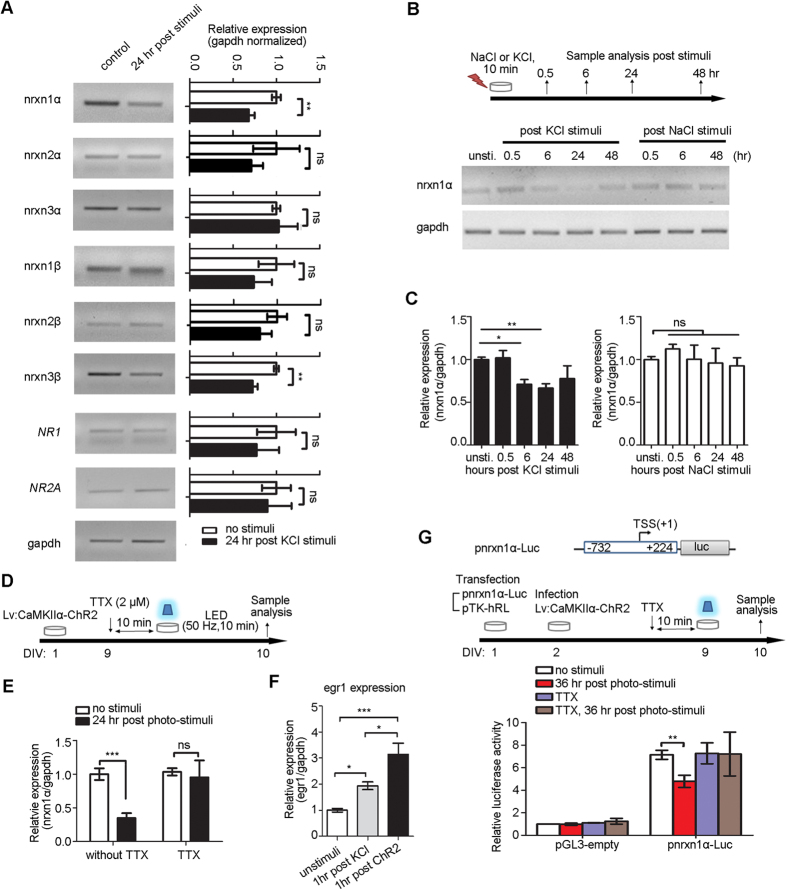
Activity-dependent repression of nrxn1α in primary cortical neuron cultures. (**A**) RT-PCR analysis of nrxns and other gene transcripts in primary cortical neuron cultures 24 hours after stimulation by a high K^+^ concentration (51 mM, 10 min) (left panel). Real-time qPCR analysis revealed a decrease in nrxn1α and nrxn3β mRNA after high K^+^ stimulation (full-length gels in Fig. S1; n = 4 biological samples; *Student’s t-test*, **P < 0.01, ns, no significance). (**B,C**) Primary cortical neuron cultures were subjected to transient stimulation by KCl or NaCl on DIV9 (**B**) (full-length gels in Fig. S1). The nrxn1α expression was analyzed using RT-PCR after stimulation (**C**) (n = 13 biological samples for KCl stimulation; n = 3 biological samples for NaCl stimulation; One-way ANOVA followed by Dunnett’s post hoc test, *P < 0.05, **P < 0.01, ns, no significance). (**D,E**) Primary cortical neuron cultures were infected by lenti-virus LV: CamKIIα-ChR2-EGFP at DIV 1 and stimulated with LED (50 Hz, 5 ms/pulse, 10 min). Expression of nrxn1α was quantified using qPCR (n = 9 for normal group, n = 3 for TTX treatment group; two -way ANOVA followed by a Bonferroni post hoc test, ***P < 0.001, ns, no significance). (**F**) Levels of egr1 expression induced by KCl stimuli (grey) or optical stimuli (black) (n = 8 for control group, n = 5 for KCl stimuli group, n = 5 for light stimuli group; *P < 0.05, one -way ANOVA followed by Tukey’s post hoc test, *P < 0.05, ***P < 0.001). (**G**) Luciferase assay revealed activity-dependent repression of nrxn1α promoter activity. The nrxn1α promoter is cloned to drive the expression of firefly luciferase in pGL3-enhancer. pTK-renilla luciferase was co-transfected as the control. Luciferase activity was measured 36 hours after optogenetic stimulation. The ratio of Firefly/Renilla luciferase activity was reported (n = 6 for normal group, n = 3 for TTX treatment group; two-way ANOVA with Bonferroni post hoc test, **P < 0.01).

We wanted to know whether the expression levels of nrxns transcripts (α-neurexins and β-neurexins) were regulated by neural activity in primary cortical neuron cultures prepared from 16-day-old ICR mouse embryos (E16, ICR strain). The expression levels of nrxns transcripts were analyzed by quantitative real-time PCR 24 hours after high K^+^ stimulation (51 mM, 10 min) in primary cortical neuron cultures on day *in vitro* (DIV) 9. The transcripts of nrxn1α and nrxn3β showed a significant reduction compared to the control (Fig. 1A; *P *< 0.05, *Student’s t-test*). In contrast, the mRNA of other nrxns, *NR1* and *NR2A*, remained unchanged (Fig. 1A). The reduction of nrxn1α mRNA was first evident at 6 hours and lasted up to 48 hours post stimuli (Fig. 1B,C; one-way ANOVA, *F*(4, 50) = 9.55, *P *< 0.001).

To further characterize the activity-dependent repression of nrxn1α, primary cortical neuron cultures (E16 ICR strain) were infected with lenti-virus (LV: CamKIIα-ChR2) and stimulated with blue light using an LED (Fig. 1D). The expression of nrxn1α showed a significant reduction 24 hours after optical stimulation (Fig. 1E). In contrast, TTX (2 μM) treatment prevented a decrease in nrxn1α mRNA in cultures 24 hours after optical stimulation, thus indicating the neuronal spiking activity reduced nrxn1α expression (two-way ANOVA, TTX treatment × light stimulation interaction: *F*(1, 22) = 4.32, *P* = 0.05; TTX treatment *F*(1, 22) = 7.10, *P* = 0.01; light stimulation *F*(1, 22) = 5.39, *P* = 0.03) (Fig. 1E).

The stimuli-induced repression of nrxn1α mRNA was different between high K^+^ treatment (Fig. 1A) and optical stimulation (Fig. 1E). Such a difference might reflect the different activity levels caused by those stimuli. Consistent with this idea, the expression level of egr1 in optical-stimulated neuronal cultures was significantly higher than that in the high K^+^-stimulated cultures (Fig. 1F; one-way ANOVA, *F*(2, 14) = 22.50, *P *< 0.05).

To test whether the reduction of nrxn1α expression was due to transcriptional activity, we cloned the promoter of nrxn1α in pGL3-enhancer luciferase reporter construct (pnrxn1α-luc). As a control, a constitutively expressing reporter, Renilla luciferase (pTK-hRL), was co-transfected with pnrxn1α-luc in primary cortical neuron cultures that were infected with LV: CamKIIα-ChR2. On DIV9, cells were stimulated with LEDs at 50 Hz, and the luciferase activity in each group was analyzed 36 hours after the stimuli application. Luciferase activity for the pnrxn1α-luc reporter showed significant repression compared to the control group (two-way ANOVA, TTX treatment × light stimulation interaction: *F*(1, 7) = 10.56, *P* = 0.01; TTX treatment *F*(1, 7) = 2.00, *P* = 0.20; light stimulation (1, 7) = 11.81, *P* = 0.01; Bonferroni post hoc tests for light stimuli in the control group, *P *< 0.01) (Fig. 1G). Such repression was blocked by TTX (Bonferroni post hoc tests for light stimuli in the TTX group, *P* > 0.05), indicating the neuronal spiking activity repressed the transcriptional activity of the nrxn1α promoter (Fig. 1G).

### Identification of the nrxn1α promoter binding proteins by the synthetic zinc finger protein

**Figure 2:**
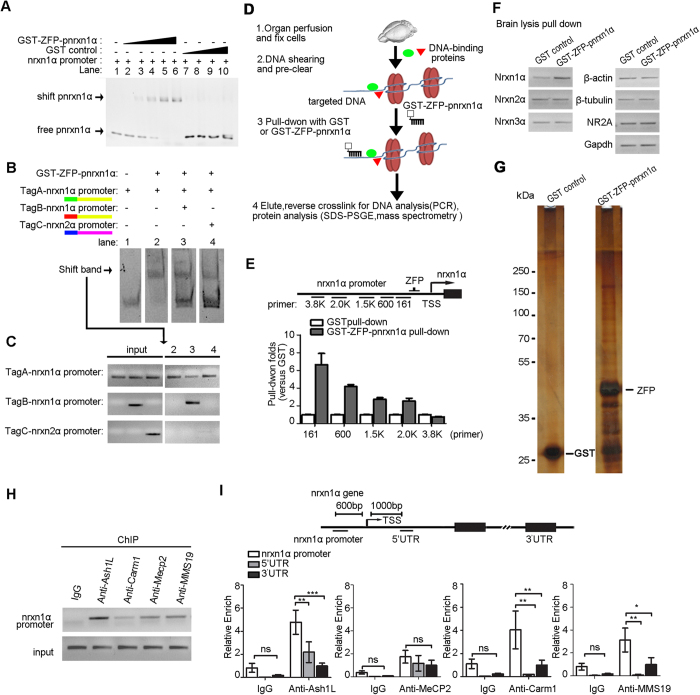
Synthetic ZFP-based chromatin purification identified Ash1L as the transcriptional regulator of nrxn1α in mouse brain. (**A**) Electrophoretic mobility shift assays were performed to test the binding capacity of the designed GST-ZFP-pnrxn1α. The PCR product of the nrxn1α promoter fragment (nrx1α promoter) was incubated with GST-ZFP-pnrxn1α or GST at the indicated concentrations. Lane 1, no protein; lane 2–6, 0.02, 0.04, 0.08, 0.12, 0.16 μM GST-ZFP-pnrxn1α, respectively; lane 7–10, 0.04, 0.08, 0.12, 0.16 μM GST, respectively (full-length gel in Fig. S2). (**B,C**) Competition assays for EMSA. The DNA probes with different paired-end tags were incubated with 0.08 μM GST-ZFP-pnrxn1α (**B**). The shift bands were subjected to semi-quantitative PCR to identify the ID of binding probes (**C**) (full-length gel in Fig. S2). (**D**) The experimental procedure of zinc finger protein-based chromatin purification. (**E,F**) PCR from purified chromatins showed specific enrichment of the promoter region of nrxn1α in GST-ZFP-pnrxn1α pull-down samples (**E**) (full-length gels in Fig. S2) and the enrichment near the TSS site of nrxn1α (**F**) (full-length gels in Fig. S2). (**G**) Proteins associated with the nrxn1α promoter were resolved by SDS-PAGE and silver staining (full-length gels in Fig. S2). (**H,I**) ChIP analysis showed the identified proteins specifically enriched at the nrxn1α promoter in the hippocampus of 3-month-old C57BL/6 mice were quantified according to the real-time PCR signal (full-length gels in Fig. S2). The relative enriched signal at the promoter region was normalized against the signal obtained at 3′UTR. (n > 3 biological samples for each group; two-way ANOVA with Bonferroni post hoc test, *P < 0.05, **P < 0.01, ***P < 0.001).

**Figure 3:**
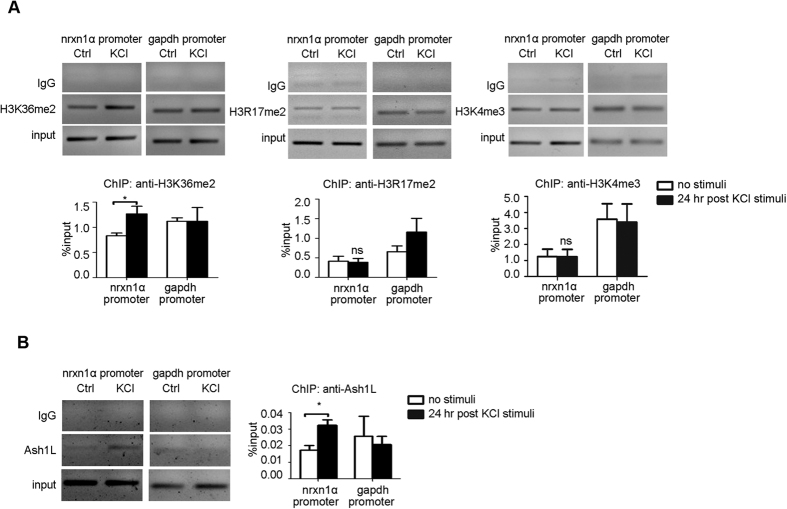
Neuronal activity recruits Ash1L and H3K36me2 to the nrxn1α promoter. (**A,B**). ChIP experiments revealed the changes of histone modifications (**A**) (full-length gels in Fig. S3; n > 3 biological samples) and Ash1L (**B**) (full-length gels in Fig. S3; n = 4 biological samples) in the nrxn1α promoter 24 hours after high K^+^ stimulation in primary neuronal cultures (E16, ICR strain). Immunoprecipitated chromatin was quantified with real-time PCR. *Student’s t-test*, *P < 0.05.

To identify the transcriptional regulators for activity-dependent repression of nrxn1α in neurons, we designed a synthetic zinc finger protein that targeted the nrxn1α promoter (GST-ZFP-pnrxn1α) to directly pull down the chromatins around nrxn1α in the mouse brain. Zinc finger proteins bind to a specific DNA sequence via the interaction between amino acids in the recognition domain of the protein and the sequence specific DNA (Table 1). To test the binding capacity of the designed GST-ZFP-pnrxn1α, an electrophoretic mobility shift assay was performed as previously reported^31^. GST-ZFP-pnrxn1α showed dose dependent binding to the DNA fragment of the nrxn1α promoter. As a control, GST alone did not show binding activity (Fig. 2A). To further test the binding specificity, the DNA fragments with different tags were used for the competition assay. Only the DNA fragment of the nrxn1α promoter sequence could compete with the TagA-nrxn1α promoter sequence to bind to the GST-ZFP-pnrxn1α protein (Figs 2B and 3C). Thus, GST-ZFP-pnrxn1α specifically binds to the nrxn1α promoter.

With the designed GST-ZFP-pnrxn1α, we pulled down the proteins binding to the nrxn1α promoter in the mouse brain (Fig. 2B). The whole brains of three 3-month-old C57BL/6 mice were fixed with formaldehyde to cross link DNA with the adjacent proteins associated with the chromatin *in vivo*. The chromatin was sheared to ~1500 base pair sections and then used to be affinity purified by the GST-ZFP-pnrxn1α collection. The DNA fragments and proteins enriched by GST-ZFP-pnrxn1α were then analyzed by PCR and mass spectrometry.

To test the specificity of the chromatins enriched by GST-ZFP-pnrxn1α pull down, elution was subjected to PCR-based analysis. The promoter region of nrxn1α was enriched in the GST-ZFP-pnrxn1α pull-down samples compared to the GST control pull-down samples (Fig. 2C). Other chromatin regions did not show such enrichment, such as the promoter of β-actin, β-tubulin, nrxn2α, nrxn3α, *NR2A, GAPDH* (Fig. 2C). Importantly, the enrichment of precipitated chromatin increased significantly near the TSS site of nrxn1α (Fig. 2D, bottom), indicating the specificity of this method.

Proteins associated with the nrxn1α promoter were enriched by the GST-ZFP-pnrxn1α pull down, detected by sliver staining, and identified by high-resolution mass spectrometry (Fig. 2E and Table 2).

### Ash1L binds to the nrxn1α promoter

Among the identified proteins, we confirmed that Ash1L, a histone methyltransferase, was associated with the nrxn1α promoter using chromatin immunoprecipitation (ChIP) in the hippocampus of 3-month-old C57BL/6 mice (two-way ANOVA, genomic loci × antibody interaction: *F*(2, 27) = 5.28, *P* = 0.01; genomic loci *F*(2, 27) = 10.89, *P* = 0.0003; anti-Ash1L *F*(1, 27) = 29.50, *P* = 0.0001; Bonferroni post hoc tests for genomic loci in the anti-Ash1L sample: promoter region vs. 5′UTR, *P *< 0.01; promoter region vs. 3′UTR, *P *< 0.001) (Fig. 2F,G). The ChIP experiments also confirmed that other proteins identified by mass spectrometry also bound to the nrxn1α promoter region in the brain, such as Carm1 (two-way ANOVA, genomic loci × antibody interaction: *F*(2, 31) = 2.55, *P* = 0.09; genomic loci *F*(2, 31) = 8.04, *P* = 0.002; anti-Carm1 *F*(1, 31) = 6.24, *P* = 0.02; Bonferroni post hoc tests for genomic loci in anti-Carm1: promoter region vs. 5′UTR, *P *< 0.01; promoter region vs. 3′UTR, *P *< 0.01), and MMS19 (two-way ANOVA, genomic loci × antibody interaction: *F*(2, 30) = 2.07, *P* = 0.14; genomic loci *F*(2, 30) = 6.15, *P* = 0.01; anti-MMS19 *F*(1, 30) = 5.54, *P* = 0.03; Bonferroni post hoc tests for genomic loci in anti-MMS19: promoter region vs. 5′UTR, *P *< 0.05; promoter region vs. 3′UTR, *P *< 0.01) (Fig. 2G). Interestingly, although MeCP2 was also enriched in the nrxn1α region, no significant difference in its binding between the promoter region and 5′UTR region was observed (two-way ANOVA, genomic loci × antibody interaction: *F*(2, 33) = 0.19, *P* = 0.83; genomic loci *F*(2, 33) = 1.05, *P* = 0.36; anti-MeCP2 *F*(1, 33) = 12.74, *P* = 0.001; Bonferroni post hoc tests for genomic loci in the anti-MeCP2: promoter region vs. 5′UTR, *P* > 0.05; promoter region vs. 3′UTR, *P* > 0.05) (Fig. 2G). Although Ash1L showed the highest enrichment in the nrxn1α region, we focused our study on Ash1L.

### Neuronal activation recruits Ash1L to the nrxn1α promoter

Ash1L is reported as a histone methyltransferase, whereas the specificity of its enzymatic activity remains unclear. Histone 3 lysine 36 di-methylation (H3K36me2) and histone 3 lysine 4 tri-methylation (H3K4me3) have both been ascribed to Ash1L^32^,^33^,^34^,^35^. We performed ChIP-qPCR experiments for H3K36me2, H3K4me3 and H3R17me2 24 hours after high K^+^ stimulation in primary neuronal cultures (E16, ICR strain). H3K36me2 showed specific enrichment at the nrxn1α promoter 24 hours after high K^+^ stimulation (Fig. 3A left; *P *< 0.05, *Student’s t-test*). Ash1L was enriched at the nrxn1α promoter after stimuli (Fig. 3B; *P *< 0.05, *Student’s t-test*). Thus, neuronal activity recruited Ash1L to the nrxn1α promoter and enriched H3K36me2 in this region.

### Ash1L mediates activity-dependent, long-term repression of nrxn1α

**Figure 4:**
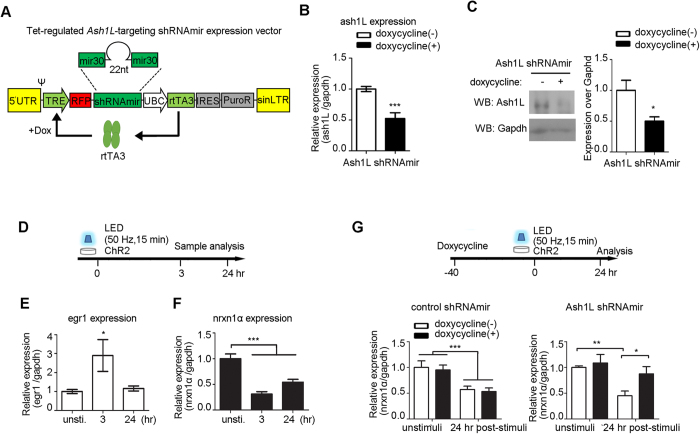
Knockdown of Ash1L reduced neuronal activity-induced repression of nrxn1α. (**Α**) Τhe design of doxycycline-inducible shRNAmir against Ash1L. The transcription of shRNAmir and TurboRFP are under the control of the tetracycline response element (TRE) promoter, which can be activated by the reverse tetracycline transactivator 3 (rtTA3) in the presence of Dox. (**B,C**) Reduction of mRNA Ash1L (n = 3 each group; *Student’s t-test*, ***P < 0.001) and protein levels (full-length blots in Fig. S4; n = 4 each group; *Student’s t-test*, *P < 0.05) 48 hours after induction in NG108-15 cells. (**D–F**) Optogenetic stimulation induced expression changes of egr1 (**E**) and nrxn1α (**F**) in differentiated NG108-15 cells. Real-time PCR was performed for quantification (n > 6 biological samples, one-way ANOVA with Dunnett’s post hoc test, *P < 0.05, ***P < 0.001). (**G**) The activity-dependent repression of nrxn1α was rescued by the Dox-induced knockdown of Ash1L knock-down cells (n > 5; two-way ANOVA with Bonferroni post hoc test, *P < 0.05, **P < 0.01, ***P < 0.001).

We next examined if Ash1L is necessary for the activity-dependent repression of nrxn1α. To knock down Ash1L, we used a doxycycline-inducible RNA interference vector (pTRIPZ) and transfected it in the NG108–15 cell line (Fig. 4A). Dox effectively reduced the Ash1L mRNA level 48 hours after induction (Fig. 4B; *P *< 0.001, *Student’s t-test*), leading to the reduction of Ash1L protein levels to 55.8% in NG cells (Fig. 4C; *P *< 0.05, *Student’s t-test*).

Next, NG108-15 cells were co-transfected with pCAG-ChR2 and shRNAmir-Ash1L and then stimulated with LEDs for 15 minutes at 50 Hz (Fig. 4D). The expression of egr1 was induced 3 hours after optical stimulation and returned to basal levels 24 hours later, thus indicating the cells were activated (Fig. 4E; one-way ANOVA followed by Dunnett’s post hoc test, *F*(2, 20) = 4.30, *P* = 0.03). Similar to primary cortical neuron cultures, the optogenetic activity-induced repression of nrxn1α (Fig. 4F; one-way ANOVA followed by Dunnett’s post hoc test: *F*(2, 27) = 27.48, *P *< 0.0001). Such activity-dependent repression was largely blocked by the Dox-induced knockdown of Ash1L (two-way ANOVA, Ash1L knockdown factor × light stimulation factor interaction: *F*(1, 23) = 2.13, *P* = 0.16; Dox-induced knockdown *F*(1, 23) = 4.87, *P* = 0.04; light stimulation *F*(1, 23) = 10.80, *P* = 0.003) (Fig. 4G, right panel). In the control (control shRNAmir) transfected cells, Dox did not induce differences in the light stimulated sample (two-way ANOVA, Dox × light stimulation interaction: *F*(1, 17) = 0.002, *P* = 0.97; Dox-induced knockdown *F*(1, 17) = 0.17, *P* = 0.69; light stimulation *F*(1, 17) = 17.35, *P* = 0.0006) (Fig. 4G).

### *Ash1L*-deficient mice brains exhibit normal morphology

**Figure 5:**
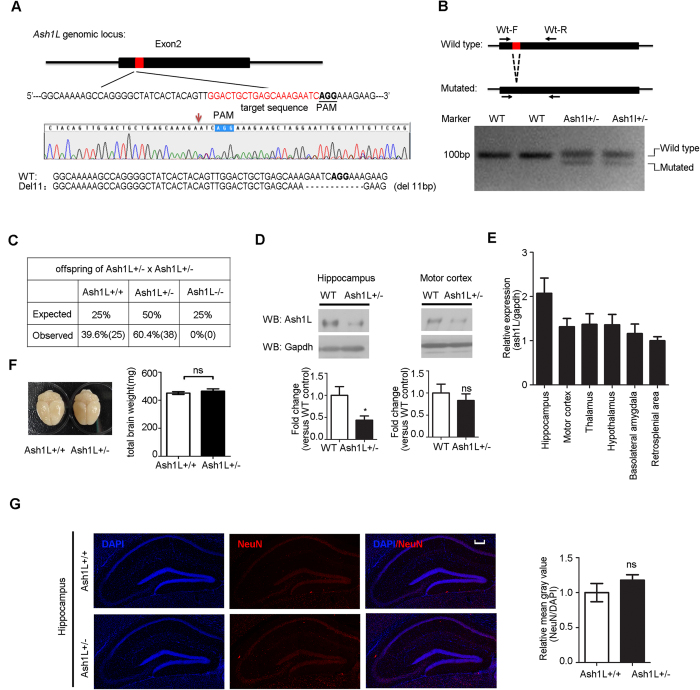
The generation of the Ash1L mutant mice. (**A**) CRISPR/Cas9-mediated mutation in the *Ash1L* locus. The top panel shows a schematic diagram of exon 2. The location of the targeted sequence is indicated in red with the corresponding PAM underlined. An indel mutation at the target locus revealed by DNA sequencing is shown in the middle panel. T-A cloning and DNA sequencing showed a targeted 11 bp deletion, which is indicated by a hyphen on the bottom panel. (**B**) A primer pair of Ash1L-Mt-F/R was used to distinguish between the wild-type allele and the 11 deletion allele (full-length gels in Fig. S5). (**C**) Genotyping of litters from *Ash1L* (−/+) intercrosses. (**D**) Western blot analysis of Ash1L in the hippocampus, motor cortex, in 3-month-old WT mice and *Ash1L* (−/+) mice (full-length blots and hypothalamus and thalamus in Fig. S5; n = 6 for WT mice, n = 9 for *Ash1L* (−/+) mice; *Student’s t-test*, *P < 0.05). (**E**) Ash1L expression in brain regions of 3-month-old WT mice. The amount of Ash1L mRNA were quantified by q-PCR relative to *GAPDH*. Data are presented as the amount relative to the retrosplenial area (n = 5). (**F,G**) *Ash1L* (−/+) mice aged 3 months showed normal brain weights (**F**) (n = 12 for WT mice; n = 13 for *Ash1L* (−/+) mice) and neuron density in the hippocampus (**G**) (n = 3 for WT mice; n = 4 for *Ash1L* (−/+) mice; blue, DAPI; red, NeuN). *Student’s t-test*, *P < 0.05, ns, no significance. Scale bar = 200 μm.

To further confirm the effect of Ash1L in activity-dependent nrxn1α repression, we generated *Ash1L*-knockout mice using the CRISPR/Cas9 system^36^. sgRNA and Cas9-coding mRNA were co-injected into C57BL/6 zygote pronuclei. We obtained 28 neonates, 4 of which showed a mutation at the target locus that was revealed by DNA sequencing. One founder was a heterozygously mutated clone with an 11 bp deletion in exon 2 leading to a null allele of ash1L (Fig. 5A). Such mutations were detectable in each animal using PCR (Fig. 5B). The homozygous offspring was absent at 1 week of age from intercrosses of the *Ash1L*(−/+) mice (Fig. 5B,C), indicating that the homozygous mutant of Ash1L had embryonic lethality. The *Ash1L*(−/+) mice were backcrossed to wild-type C57BL/6 mice for more than 5 generations to eliminate any potential off-target effects. Meanwhile, the expression of the Ash1L protein was significantly reduced in the hippocampus of 3-month-old *Ash1L* (−/+) mice (Fig. 5D, right panel; *P *< 0.05, *Student’s t-test*). Furthermore, the transcription of Ash1L in the hippocampus was relatively high compared to other brain areas (Fig. 5E).

The 3-month-old *Ash1L*(−/+) mice did not show any significant abnormalities in brain size (Fig. 5F). The neuron density and overall morphology in the hippocampus of 3-month-old *Ash1L*(−/+) mice were comparable to wild-type C57BL/6 littermates (Fig. 5G).

**Figure 6:**
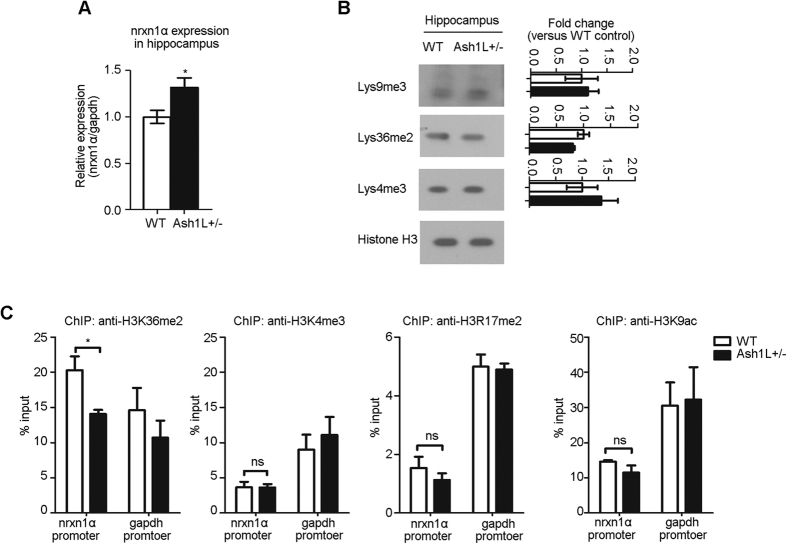
Increased nrxn1 expression and reduced H3K36me2 at the nrxn1α promoter in the hippocampus of *Ash1L* (−/+) mice. (**A**) Real-time PCR showed up-regulation of nrxn1α mRNA in the hippocampus of *Ash1L* (−/+) mice aged 3 months (n = 4 each group). (**B**) Samples from the hippocampus showed no changes in histone methylation levels in *Ash1L* (−/+) mice (full-length blots in Fig. S6; n = 3 each group). (**C**) ChIP was performed on the nrxn1α promoter using antibodies for H3K36me2, H3K4me3, H3R17me2 and H3K9ac in the hippocampus of wild-type and *Ash1L* (−/+) mice aged 3–4 months (n = 3). *Student’s t-test*, *P < 0.05.

Consistent with the neuronal cultures studies, *Ash1L*(−/+) mice exhibited up-regulation of nrxn1α mRNA in the hippocampus (Fig. 6A; *P *< 0.05, *Student’s t-test*), suggesting that Ash1L is involved in the regulation of nrxn1α transcription. Such regulation might not be due to the overall changes in the epigenetic landscape, as the H3K36me2 and H3K4me3 in the hippocampus were not significantly changed (Fig. 6B). In the hippocampus of *Ash1L*(−/+) mice (3 months of age), the ChIP experiments revealed that the enrichment of H3K36me2 at the nrxn1α promoter region was significantly decreased (Fig. 6C; *P *< 0.05, *Student’s t-test*). In contrast, other histone modifications in the nrxn1α promoter were unchanged, including H3K4me3, H3R17me2 and H3K9ac (Fig. 6C). Thus, Ash1L was essential for the regulation of H3K36me2 at the nrxn1α promoter, but not the total level of H3K36me2.

### Activity-dependent repression of nrxn1α is abolished in *Ash1L*−/− cortical neuronal cultures

**Figure 7:**
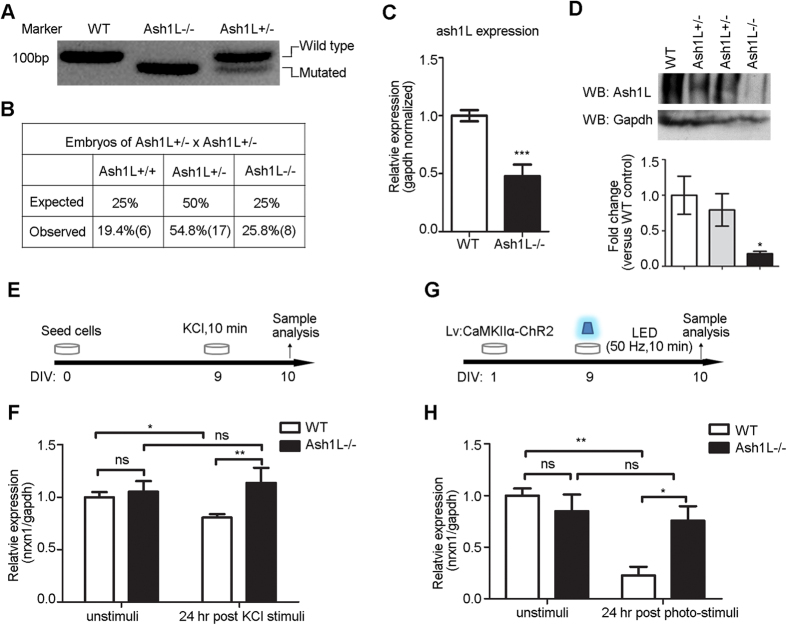
Ash1L mediated the activity-dependent repression of nrxn1α. (**A,B**) PCR genotyping (**A**) (full-length gels in Fig. S7) and genotypes (**B**) of E14 embryos from *Ash1L* (−/+) intercrosses. (**C**) The levels of ash1L mRNA were measured by real-time PCR in wild-type neurons and *Ash1L*−/− neurons (n = 7 biological samples; *Student’s t-test*, ***P < 0.001). (**D**) Western blot revealed Ash1L protein levels in WT, *Ash1L*−/+ and *Ash1L*−/− neuron cultures (n = 3 each group; *Student’s t-test*, *P < 0.05). (**E**,**F**) Real-time PCR analysis revealed the high K^+^-induced repression of nrxn1α was absent in *Ash1L*−/− neurons (n > 3 biological samples; two-way ANOVA with Bonferroni post hoc test, *P < 0.05, **P < 0.01). (**G,H**) Real-time PCR analysis revealed the optogenetic stimuli-induced repression of nrxn1α was absent in *Ash1L*−/− neurons (n = 4 biological samples; two-way ANOVA with Bonferroni post hoc test, *P < 0.05, **P < 0.01).

To confirm the role of Ash1L in the activity-dependent repression of nrxn1α, we further identified a few homozygous embryos from E14 by intercrossing of the *Ash1L*(−/+) mice (Fig. 7A,B). *Ash1L*−/− primary cortical neuron cultures derived from embryos (E14, *Ash1L*(−/+) mouse) showed low levels of Ash1L mRNA (Fig. 7C; *P *< 0.001, *Student’s t-test*), which might be due to the mRNA surveillance mediated by nonsense-mediated decay. The 11 bp deletion in exon 2 might lead to instability of missense transcripts in *Ash1L*−/− culture neurons. When tested at the protein levels, *Ash1L*−/− primary cortical neuron cultures showed almost no expression of the intact Ash1L protein (Fig. 7D; *P *< 0.05, *Student’s t-test*). The cultured neurons showed no activity-dependent repression of nrxn1α 24 hours after high K^+^ stimulation (two-way ANOVA, genotype × KCl stimulation interaction: *F*(1, 16) = 4.04, *P* = 0.06; genotype *F*(1, 16) = 7.65, *P* = 0.01; KCl stimulation *F*(1, 16) = 0.63, *P* = 0.44; Bonferroni post hoc tests for high K^+^ stimulated culture neurons: wild-type high K^+^ stimuli vs. *Ash1L*−/− high K^+^ stimuli, *P *< 0.01) (Fig. 7E,F). Consistent with LV: CamKIIα-ChR2 being expressed in neurons 24 hours after LED stimulation (50 Hz, 10 min), *Ash1L*−/− primary neuronal cultures also showed no activity-dependent repression of nrxn1α (two-way ANOVA, genotype × light stimulation interaction: *F*(1, 11) = 7.20, *P* = 0.02; genotype *F*(1, 11) = 2.20, *P* = 0.17; light stimulation *F*(1, 11) = 11.47, *P* = 0.006; Bonferroni post hoc tests for light stimulation in the two genotypes culture neurons: wild-type control vs. wild-type light stimuli, *P *< 0.01; *Ash1L*−/− control vs. *Ash1L*−/− light stimuli, *P* > 0.05; wild-type light stimuli vs. *Ash1L*−/− light stimuli, *P *< 0.05)(Fig. 7G,H). These results indicated that Ash1L mediates the neuronal activity-dependent repression of nrxn1α.

## Discussion

We have identified a unique process of activity-dependent repression in the gene transcription of nrxn1α. Furthermore, using a newly developed tool to isolate chromatin-associated proteins at specific genomic loci, we identified an epigenetic regulator required for the neuronal activity-dependent long-lasting repression of nrxn1α. We found that transient membrane depolarization recruits a histone modifier, Ash1L, to the nrxn1α promoter to enrich H3K36me2 in this region, leading to a long-term repression of nrxn1α transcription.

Since the finding of neuronal activity-dependent gene transcription in the late 1980s, many activity-dependent genes have been identified, which showed essential functions in regulating neuronal plasticity^37^. Most of the studies have focused on the activity-dependent increase of expression, and very few studies have examined activity-dependent repression.

In fact, activity-dependent repression and elimination are important processes for normal brain function. A key step in the refinement of neuronal wiring during the late stage of development in the postnatal brain is mediated by the activity-dependent elimination of synapses^38^,^39^,^40^. Activity-dependent transcription factors, such as MEF2, may regulate synapse elimination and suppression^41^. Such a process coordinates the expression of a broad program of gene expression, including *Bdnf*^42^, *Arc* and *Homer1*^43^. Here, we extended this finding by showing that neurexin-1α, an essential protein for synapse formation, undergoes activity-dependent repression. Furthermore, by discovering the novel role of Ash1L in this process, we suggested that the epigenetic regulation is involved in the repression of synapse formation, implicating the function of epigenetic regulation in the long-term maintenance of memory circuits^18^.

Some methods have been established to identify the molecules bound to specific genomic loci *in vivo*^44^,^45^,^46^. Insertional chromatin immunoprecipitation (iChIP) was shown to be useful for this purpose. By inserting repeats of exogenous binding sites in the genome, this method utilizes an engineered DNA-binding protein to enrich the targeted genome loci to purify proteins associated with the inserted loci. However, the insertion of an exogenous DNA sequence might affect the transcriptional regulation of the endogenous genes. Here, we developed a new method. By using synthetic zinc finger proteins, we were able to enrich the targeted chromatin as the native state *in vivo.* Although the sensitivity of this method still needs to be improved, we have successfully identified a novel transcriptional regulator for neurexin-1α. Our screening also detected MeCP2 as a regulator for the neurexin-1α expression. The regulation of neurexin-1α expression by MeCP2 has been reported^47^, suggesting the specificity of this method.

Ash1 was originally identified as one of the epigenetic regulators in Drosophila. The *ash1* gene encodes a member of the trithorax group (TrxG) of proteins that maintain active transcription by competing with Polycomb proteins^48^,^49^. The mammalian ortholog, Ash1L, acts as histone methyltransferase targeting H3K36me2^33^,^50^,^51^. However, H3K36me2 also recruits histone deacetylase to repress spurious transcripts within the gene body^16^,^17^. Similar repression mechanisms might also take place in the H3K36me2-dependent repression of the alternative promoter in nrxn1α. Therefore, Ash1L could both enhance and repress gene expression, depending on the genomic environments on the regulated gene targets^16^,^52^.

Ash1L is widely expressed in multiple organs and enriched in the brain^51^,^53^,^54^. Its expression is enriched in the hippocampus so that the protein level of Ash1L in the hippocampus is sensitive to the genomic deletion in one allele. However, the roles of Ash1L in the brain remain poorly understood. Here, we identified a novel role of Ash1L in activity-induced repression of neurexin-1α expression. Further studies on the regulation of Ash1L and its role in the adult brain might help to improve our understanding of the roles that epigenetic modifications play in regulating brain function, especially in activity-dependent network rewiring. Such studies might also uncover the neural basis of cognitive diseases, such as autism spectrum disorder.

## Materials and Methods

### Plasmid Construction

The creation of a six-finger Zinc Finger protein targeting nrxn1α promoter (GST-ZFP-pnrxn1α) was carried out according to a previously described procedure^55^. DNA sequences encoding the first zinc finger were digested with *Xho*I and *Spe*I, and subsequently cloned into a *Xho*I/*Spe*I-digested pSCV vector to create one-finger ZF insertion pSCV-1ZF. The last five zinc finger-encoding sequences were digested with *Xma*I and *Spe*I and then cloned into *Age*I/*Spe*I-digested pSCV-1ZF successively to create six-finger ZF insertion pSCV-6ZF. Finally, the 6ZF encoding sequence was digested with *Xma*I/*Spe*I and cloned into *Xma*I/*Spe*I-digested pGEX-4T-1, which has a modified multiple cloning site, creating pGEX-6ZF. Insertion was confirmed by sequencing.

The 900 bp nrxn1α promoter was cloned into the *Kpn*I and *Xho*I site of pGL3-enhancer luciferase reporter vector to create pnrxn1-Luc plasmid. The primers used to amplify nrxn1α promoter from mouse genomic DNA are listed in Table 3.

The sequence of oligonucleotides for short hairpin RNA (shRNAmir) was designed on the web: http://katahdin.cshl.org/siRNA/RNAi.cgi?type=shRNA. The oligonucleotide sequence was as follows: 5′-ACCTCATTATGTCCCAGACAAC-3′. The sequence was cloned into doxycycline-inducible pTRIPZ (Open Biosystems) lenti-viral vectors between its *EcoR*I and *Xho*I sites. Cells were treated with doxycycline (1 μg/ml) to induce expression of shRNAmirs.

### Glutathione S-transferase (GST)-tagged zinc finger protein expression and purification

For synthetic ZFP-pnrxn1α purification, *E. coli* BL21 (DE3) transformed with pGEX-6ZF were grown in Luria-Bertani (LB) medium supplemented with ampicillin (100 μg/ml) at 37 °C, and then IPTG (final concentration, 1 mM) was added when the A_600_ of the culture reached 0.6. After incubation for 12 hours at 16 °C, the bacteria were harvested, re-suspended in lysis buffer (20 mM Tris-HCl, 150 mM NaCl, pH 8.0), and lysed via sonication. Lysates were cleared by centrifugation at 13000 rpm for 20 min. The GST-ZFP-pnrxn1α was purified using a Pierce GST spin purification kit (Thermo Scientific) according to the manufacturer’s instructions. Ultrafiltration centrifugation (Millipore) was conducted to remove GSH and concentrate protein. Purified protein was assessed by SDS-PAGE, and the concentration was quantified using the Bradford method (Transgen).

### Electrophoretic mobility shift assay

The nrxn1α promoter probe was amplified by standard PCR from the C57BL/6 mouse genome. Purified proteins were incubated with probes at 37 °C for 30 min in buffer (50 mM Tris-HCl, 150 mM NaCl, 5% glycerol, 1 mM dithiothreitol, 0.1% NP-40, pH 8.0), followed by electrophoresis in 8% native polyacrylamide gels in 0.5 × TBE buffer, at 80 V for 2 h. The gels were stained with ethidium bromide and visualized under ultraviolet transillumination. For EMSA competition assay, DNA fragments with paired-end tags were prepared by PCR amplification. A 15-fold excess of competitor was added to the incubation system. The gels containing the shift DNA-protein complexes were purified followed by PCR amplification against the paired-end tags.

### Chromatin pull-down assay

C57BL/6 mice at the age of 3 months were deeply anesthetized with an intraperitoneal injection of 2% sodium pentobarbital (400 mg/kg body weight) and subsequently perfused with 4% paraformaldehyde solution in 0.1 M phosphate buffer (pH 7.4). The brains were removed rapidly, and 1-mm-thick coronal sections were prepared using brain matrix. Fresh tissue from the cortex and hippocampus was cut into small pieces, homogenized, cross-linked with 4% formaldehyde for 15 min, and quenched with 0.125 M glycine. Cells were fragmented by sonication (Micro-tip, Branson sonicator). The chromatin segments were collected by centrifugation at 16000 g for 15 min at 4 °C and incubated with purified GST-ZFP-pnrxn1α for 2 hours. Glutathione agarose resin (Thermo Scientific) was added for 40 min, and the chromatin segments were then washed with washing buffer until the absorbance at 280 nm was stabilized at the baseline level. Bound complexes were eluted in 50 mM Tris-HCl containing 15 mM glutathione. Protease inhibitor phenylmethyl sulfonyl fluoride (1 μM, PMSF) was added to all buffers. Elution with 200 μl was reversed at 65 °C with 8 μl of NaCl (5 M), 2 μl of RNaseA (20 mg/ml) and 1 μl of proteinase K (10 mg/ml) for at least for 6 hours. DNA was purified using the phenol/chloroform extraction and ethanol precipitation method. The relative enrichment ratios within 3.8 kb regions upstream of TTS were calculated by using GST-ZFP-pnrxn1α pull-down signals normalized to GST control signals. The primers are listed in Table 3.

### Western blot analysis and silver staining of SDS-PAGE

For western blot analysis, the samples were loaded on SDS-PAGE and then transferred to nitrocellulose membranes (Bio-Rad). After blocking with blocking buffer (5% non-fat dry milk and 0.1% Tween-20 in PBS) for 1 hour at room temperature, membranes were incubated with probed antibodies overnight at 4 °C. Membranes were washed three times, incubated with HRP conjugated secondary antibodies for 1 hour at room temperature, and the signals were detected with chemiluminescence solution (Thermo Scientific). The following antibodies were used: antibody specific to Ash1L (Novus Biologicals), H3K9me3 (Abcam), H3K4me3 (Abcam), H3K36me2 (Abcam), Histone3 (Santa Cruz Biotechnology), and GAPDH (Cell signaling).

Silver staining was performed using a modified protocol of Mortz^56^. Briefly, gels were fixed with 10% acetic acid/40% methanol for 20 min and then washed with deionized distilled water. After incubation in 0.2% Na_2_S_2_O_3_ for 30 min, gels were washed 3 times again and incubated in 0.25% AgNO_3_ for 20 min. Gels were developed with 0.04% paraformaldehyde and 2.5% Na_2_CO_3_ until the desired staining had occurred. Development was stopped by the addition of 5% acetic acid.

### Identification of Nrxn1 promoter associated proteins by mass spectrometry

GST**-**ZFP-pnrxn1α pull-down samples were separated on SDS-PAGE gel. Each gel lane, except zinc finger protein, was entirely sliced into 4 bands, reduced with 10 mM dithiothreitol, and alkylated with 55 mM iodoacetamide. In-gel digestion was then carried out with sequence-grade modified trypsin (Promega) in 50 mM ammonium bicarbonate at 37 °C overnight. The peptides were extracted twice with 0.1% trifluoroacetic acid in 50% acetonitrile aqueous solution for 30 min. High-performance liquid chromatography–tandem mass spectrometry (HPLC-MS/MS) analysis was performed as described previously^57^.

### Cell culture, transfection and optogenetic stimulation

NG108-15 cells were maintained in Dulbecco’s Modified Eagle Medium (Life Technologies) with 10% fetal bovine serum (Gibco), 10 mM hypoxanthine, 0.1 mM aminopterin, 1.6 mM thymidine, 100 U/ml penicillin and 100 mg/ml streptomycin in a humidified incubator equilibrated with 5% CO_2_ in air at 37 °C. Passages 4 through 20 were used for experience. NG108-15 cells were differentiated by allowing the cells to reach approximately 60% confluence and then replacing the serum-free neuronal medium (neurobasal medium (Life Technologies) containing 2% B27 supplement (Gibco), 2 mM glutamax (Gibco) and penicillin/streptomycin (Gibco)) for at least 36 hours. Cells were transfected with cationic lipids VigoFect (Vigorous Biotechnology).

Primary cortical neuron cultures were prepared from embryonic day 16 (E16) for ICR mice and from E14 for *Ash1L* (−/+) intercrosses. Cells were seeded onto culture dishes at a density of 5 × 10^5^ per square centimeter and grown in serum-free neuronal medium. After DIV 1, cells were transfected using Lipofectamine 2000 reagent (Invitrogen) according to the manufacturer’s protocol.

The optogenetic method used to perform the light-induced stimulation was described previously^58^. In brief, an array of LED lamps (~470 nm, 3.0–3.2 V, 1,000–1,200 mcd/lamp, 25 mW) were placed 10 cm above the culture dish. LED lamps were controlled by the SCM control electric circuit (single chip Micyoco; specifically, AT89S52 and AT89C52). Light pulses were given at 5 ms/pulse at 50 Hz. Primary cortical neuron cultures were infected with pLenti-CaMKIIα-hChR2 (H134R)-EYFP-WPRE viruses (LV: CamKIIα-ChR2) (Neuron Biotech Co., Shanghai) on DIV1. Optical stimulation was carried out on DIV9. For synaptic activity inhibition, tetrodotoxin (TTX, 2 μM) was added 10 min before stimulation. NG108-15 cells were transfected with pCAG-ChR2 plasmids for optical stimulation. All cells were kept in a dark box after transfection.

For KCl depolarization, primary cortical neuron cultures were treated with culture medium containing 51 mM KCl for 10 min on DIV9, followed by washing and medium exchange with fresh neuronal medium (without KCl).

### RNA extraction, reverse transcription, and quantitative real-time PCR

Total RNA was isolated using TRIzol reagent (Invitrogen) according to the manufacturer’s protocol, and 1.5 μg RNA was used for reverse transcription by the one step first strand cDNA synthesis kit (Transgen). Quantitative real-time PCR (qRT-PCR) was performed using SYBR Green PCR master mix (Bio-Rad) in a CFX96 machine (Bio-Rad). The relative fold-change in each mRNA expression was calculated using the ddCt method relative to the expression of *GAPDH*. The primers used for qRT-PCR are listed in Table 3.

### Cell transfection and Luciferase assays

Primary cortical neuron cultures from E16 for ICR mice were seed in a 24-well plate. DNA plasmids were delivered into cells 24 hours after plating. LV: CamKIIα-ChR2 infection was carried out on DIV2. Luciferase activities were measured 36 hours after optogenetic stimulation using Dual-Luciferase Reporter Assay System (Promega).

### Chromatin immunoprecipitation assay

The chromatin immunoprecipitation (ChIP) was performed according to online protocols provided by Miltonic Biotec with some modifications. Primary neuron cultures from E16 for ICR mice were cross-linked with 1% formaldehyde for 5 min. 3-month-old C57BL/6 mice were perfused with 4% formaldehyde, and dorsal hippocampus was homogenized, fixed in 2% formaldehyde for 15 min. Cells were quenched with 0.125 M glycine, washed with cold phosphate buffer (0.1 M, pH7.4) and then lysed with SDS-lysis buffer (1% SDS, 10 mM EDTA, 50 mM Tris-HCl, pH = 8.1) complemented with protease inhibitor cocktail. DNA fragmentation was performed with a Branson sonicator with six cycles of 15 sec on and 30 sec off. The chromatin solution was cleared by Protein G Dynabeads (Invitrogen) and then incubated with antibodies at 4 °C overnight. Protein G beads (20 μl) were added for 2 hours. The complexes were washed with low-salt immune complex wash buffer (0.1% SDS, 1% TritonX-100, 2 mM EDTA, 20 mM Tris-HCl (pH8.1), 150 mM NaCl), high-salt immune complex wash buffer (0.1% SDS, 1% TritonX-100, 2 mM EDTA, 20 mM Tris-HCl (pH8.1), 500 mM NaCl), LiCl immune complex wash buffer (0.25 M LiCl, 1% NP-40, 1% sodium deoxycholate, 1% TritonX-100, 1 mM EDTA, 10 mM Tris-HCl (pH8.1)) and TE buffer (1 mM EDTA (pH8.0), 10 mM Tris-HCl, 50 mM NaCl). The immunoprecipitants were eluted from the beads by elution buffer (1% SDS, 0.1 M NaHCO_3_) at 65 °C with vortex-mixing. The materials were reversed at 65 °C with 8 μl of NaCl (5 M), 2 μl of RNaseA (20 mg/ml) and 1 μl of proteinase K (10 mg/ml) for at least for 6 hours. DNA was purified using the phenol/chloroform extracton and ethanol precipitation method for PCR analysis. The enrichment of sequences in DNA subjected to ChIP was quantified by real-time PCR. The primers used for ChIP are listed in Table 3.

### Microinjection for generation of *Ash1L*-deficient mice

CRISPR/Cas9 system was employed to generated *Ash1L*-deficent mouse. Target sequence within the second exon of ash1L was chosen according to the sgRNA recognition guidelines described previously^59^,^60^. Three sgRNAs were designed. According to the initial tests, we choose one of the sgRNAs for the injection. *In vitro* transcription of customized sgRNAs was performed using a RiboMAX Large Scale RNA Production Systems-T7 Kit (Promega). Cas9-mRNA was synthesized *in vitro* using a mMESSAGE mMACHINE T7 Ultra Kit (Life Technologies). The sgRNA and Cas9-coding mRNA were mixed to final concentrations of 50 ng/μl and 250 ng/μl, respectively. Injection of C57BL/6 zygotes pronuclei was performed with an established setup at the Laboratory Animal Facility at the Tsinghua University. 1-week-old founder mice were identified by PCR, using template of DNA isolated form tail biopsies. The primers of Ash1L-Wt-F/R, which was used for genotype, are listed in Table 3.

### Immunohistochemistry

Immunohistochemical analysis was performed as described previously^11^. Anti-NeuN antibody (Abcam) was used at a dilution of 1:500. Confocal images (1 μm) were scanned and subjected to three-dimensional reconstruction. Brain sections with the strongest intensity were scanned first. All other images included in the analysis were scanned with the same settings (Zeiss LSM780). ImageJ software (National Institutes of Health, Bethesda, MD, USA) was used to calculate the mean neuronal density.

### Ethics statement

All animals were kept in animal research facility of Tsinghua University. The Laboratory Animal Facility at the Tsinghua University is accredited by AAALAC (Association for Assessment and Accreditation of Laboratory Animal Care International). All experimental protocols involving mice were approved and conducted in accordance with the guidelines by the Institutional Animal Care and Use Committee (IACUC) of Tsinghua University.

### Data analysis

The statistical significance between two groups was determined by *Student’s t-test*. One-way ANOVA followed by Dunnett post hoc test or Tukey post hoc test for three or more groups. Two-way ANOVA accompanied by a Bonferroni post hoc test was used for multiple comparisons using Prism 6 (Graphpad Software Inc., La Jolla, CA, USA). All data are presented as mean ± SEM; *P* values of less than 0.05 were considered statistically significant (*P < 0.05, **P < 0.01, ***P < 0.001).

## Additional Information

**How to cite this article**: Zhu, T. *et al*. Histone methyltransferase Ash1L mediates activity-dependent repression of neurexin-1α. *Sci. Rep.* **6**, 26597; doi: 10.1038/srep26597 (2016).

## Supplementary Material

Supplementary Information

## Figures and Tables

**Table 1:** Summary of synthetic zinc finger protein.

**Target sequence (5′-3′)**	**Finger 1**	**Finger 2**	**Finger 3**	**Finger 4**	**Finger 5**	**Finger 6**
agaggcggtggtgaagcc	DCRDLAR	QKSNLIR	WPSNLTR	WPSNLTR	DPGHLVR	QLAHLRA

Synthetic zinc finger protein GST-ZFP-pnrxn1α that targeting the nrxn1α promoter is shown. The target sequence in the nrxn1α promoter is listed in the 3′ to 5′ direction. The amino acid sequence of each zinc finger for binding helices is also listed.

**Table 2:** Histones and epigenetic regulators identified by GST-ZFP-pnrxn1α pull-down analysis to be enriched by >2-fold with nrxn1promoter chromatin.

**Accession**	**Description**	**Enrich fold (vs. GST)**
IPI00229544.3	Histone H2A type 1-H	69.5
IPI00228616.5	Histone H1.1	61.8
IPI00623951.3	Histone H2A type 2-B	41.2
IPI00331734.5	Histone H2A.Z	28.3
IPI00230133.5	Histone H1.5	28.3
IPI00124518.3	Hist2h2bb protein	25.7
IPI00230730.4	Histone H3.2	20.6
IPI00314240.5	Isoform HMG-I of High mobility group protein HMG-I/HMG-Y	15.4
IPI00223714.5	Histone H1.4	12.6
IPI00223713.5	Histone H1.2	12.4
IPI00407339.7	Histone H4	11.5
IPI00131063.4	Isoform A of Methyl-CpG-binding protein 2	7.7
IPI00886201.1	Histone H3	6.8
IPI00954313.1	high mobility group protein HMG-I/HMG-Y isoform c	5.1
IPI00756676.3	breast carcinoma-amplified sequence 1 homolog isoform 2	5.1
IPI00421169.2	Protein arginine N-methyltransferase 6	3.8
IPI00279931.1	Isoform 2 of Histone-arginine methyltransferase CARM1	3.8
IPI00223713.5	Histone H1.2	3.8
IPI00553465.4	Probable histone-lysine N-methyltransferase ASH1L	3.5

**Table 3:** Primers list.

**Purpose**	**Primer name**	**Sequence**
pnrxn1-Luc clone	pnrxn1-luc-F	ATGGGTACCCCTGGTGGGTTGCTCTTAAAGTC
	pnrxn1-luc-R	TAGCTCGAGCCCAAATTGAGCTCCTTTACCTG
EMSA Tag-DNA and paired-end tag primers	Tag A-nrxn1α promoter F	GTAAAACGACGGCCAGT CGCCCTTCTCTTTCAGAAGGACAG
	Tag A-nrxn1α promoter R	CAGGAAACAGCTATGAC GAGGAAGGGAAGAACATGGAACAG
	Tag B-nrxn1α promoter F	CCTACACGACGCTCTTCCGATCT CGCCCTTCTCTTTCAGAAGGACAG
	Tag B-nrxn1α promoter R	GCGGTTCAGCAGGAATGCCGAG GAGGAAGGGAAGAACATGGAACAG
	Tag C-nrxn1α promoter F	GACGGCATTTTCGTTCTTATTAG GGGTGTGGGATCCAACAACCCAAC
	Tag C-nrxn1α promoter R	GATCATAAAAGTATTAAAGTTC GGCATCCGAGCCCTTTCCCACCTC
	Tag A-F	GTAAAACGACGGCCAGT
	Tag A-R	CAGGAAACAGCTATGAC
	Tag B-F	CCTACACGACGCTCTTCCGATCT
	Tag B-R	GCGGTTCAGCAGGAATGCCGAG
	Tag C-F	GACGGCATTTTCGTTCTTATTAG
	Tag C-R	GATCATAAAAGTATTAAAGTTC
Pull down PCR	Nrxn1α-160-F	AAGGGGCTGCAGCTGCCAGCTGA
	Nrxn1α-160-R	CGTGACGCCGGGTGCTGTCCTTC
	Nrxn1α-600-F	TCCTGCCTGCCCCTGCATTC
	Nrxn1α-600-R	GGGCGGGGGTACCTGAGAAG
	Nrxn1α-1.5K-F	GAAACTCACACCCACCCAATCCG
	Nrxn1α-1.5K-R	TTTCCATCCAGAAAAACCAGAAC
	Nrxn1α-2.0K-F	GGCCCTTGGTTCTGACCCTGTC
	Nrxn1α-2.0K-R	TCCCCCAACATATCTATGGCCTG
	Nrxn1α-3.8K-F	AAGAGGTCAGGGGCAATCCAAC
	Nrxn1α-3.8K-R	TCACCAATGGCAAAAGTGAAGC
	Nrxn2α promoter-F	GGGTGTGGGATCCAACAACCCAAC
	Nrxn2α promoter-R	GGCATCCGAGCCCTTTCCCACCTC
	Nrxn3α promoter-F	TTTCCAAAAATGTGCTGACATAGC
	Nrxn3α promoter-R	TGGAGGTACTTCATACAGGGCAGG
	β-actin promoter-F	CCACTGGGGCTCGCCCTATGCTTG
	β-actin promoter-R	CCACTGGGGCTCGCCCTATGCTTG
	NR2A promoter-F	GGGTTGCTGCGTTGCGTCTCGCTG
	NR2A promoter-R	CTAGGGCACGCTTCTGCTGCGGTC
	β-tubulin promoter F	CACACTACCGCAGTCTTCACGCTC
	β-tubulin promoter R	GACGGTCTTGGGGGCTGGACTATG
	Gapdh promoter-F	ATCCTGTAGGCCAGGTGATG
	Gapdh promoter-R	AGGCTCAAGGGCTTTTAAGG
RT-PCR	Nrxn1α-RT-F:	CCAGCACAACCTGCCAAGAGGATTC
	Nrxn1α-RT-R	TGGGGCGGTCATTGGGAGGCCAC
	Nrxn1β-RT-F	TCACCAGCATCCTTGCGAGGCGGAC
	Nrxn1β-RT-R	TGGGGCGGTCATTGGGAGGCCAC
	Nrxn2α-RT-F	CTACCTTCTGCTGGACATGGGCTCC
	Nrxn2α-RT-R	GCGTGCTGCGGCTGTTCACA
	Nrxn2β-RT-F	GTCTCGTCCAGCCTCAGCACCACC
	Nrxn2β-RT-R	CGTGTACTGGGCCGGTCATTGGGA
	Nrxn3α-RT-F	AGCGTCCCTGTGAAAATGGTG
	Nrxn3α-RT-R	GATGGGGTTCTGGGACAGGTC
	Nrxn3β-RT-F	GACTTGGCGACTTCCTCCAGC
	Nrxn3β-RT-R	GCCAGTTGTCCACCTGAAGTG
	NR1-RT-F	TCATCCTGCTGGTCAGCGATGAC
	NR1-RT-R	AGAGCCGTCACATTCTTGGTTCCTG
	NR2A-RT-F:	TGATGAACCGCACTGACCCTAAG
	NR2A-RT-R	GGAAGAACGTGGATGTCGGATC
	Ash1L-RT-F	AAGAGCCCAGTGAAAACATCAAC
	Ash1L-RT-R	CCTCCTCCTGCTTCTGATGGA
	Egr1-RT-F	GAGCGAACAACCCTATGAGC
	Egr1-RT-R	AGCGGCCAGTATAGGTGATG
	Gapdh-RT-F	CTCCCCCCCACCATCCGGGTTCCT
	Gapdh-RT-R	CCTTGACTGTGCCGTTGAATTTGC
ChIP-PCR	Nrxn1 promoter-ChIP-F	TCCTGCCTGCCCCTGCATTC
	Nrxn1 promoter-ChIP-R	GGGCGGGGGTACCTGAGAAG
	Nrxn1 5 UTR-ChIP-F	CAAGCTGAGAATGGCCTCAAAGCAC
	Nrxn1 5 UTR-ChIP-R	CCCACTCCTGTGCCAAACCCAC
	Nrxn1 3′UTR-ChIP-F	TGGTTCATCCCTAAGAAGTCAAC
	Nrxn1 3′UTR-ChIP-R	TGGATAAATTGGCCTTGCAACAG
	Gapdh promoter-ChIP-F	ATCCTGTAGGCCAGGTGATG
	Gapdh promoter-ChIP-R	AGGCTCAAGGGCTTTTAAGG
*Ash1L*(+/−) genotyping	Ash1L-Wt-F	TTAGGCAAAAAGCCAGGGGCTATC
	Ash1L-Wt-R	CTCTTTATTCACTAGCCCTGGAAC
