# Supplementary material for: Histone methyltransferase Ash1L mediates activity-dependent repression of neurexin-1α
Source: Sci Rep. 2016 May 27;6:26597. doi: 10.1038/srep26597 (PMC4882582; doi:10.1038/srep26597)
Supplement: Supplementary Information [file srep26597-s1.pdf]

Histone methyltransferase Ash1L mediates activity-dependent repression of neurexin-1α

Tao Zhu[#], Chen Liang[#], Dongdong Li, Miaomiao Tian, Sanxiong Liu, Guanjun Gao, Ji-Song Guan*

MOE Key Laboratory of Protein Sciences, Tsinghua-Peking Center for Life Sciences, School of Life Sciences, Center for Brain-Inspired Computing Research, IDG/McGovern Institute for Brain Research, Tsinghua University, Beijing 100084, China.

* Please address correspondence to:

Ji-Song Guan, Ph.D.

School of Life Sciences

Medical Science Building, Room D206

Tsinghua University

Beijing, 100084, P.R.China

Tel: 86-10-62785905

Fax: 86-10-62785905

Email: jsguan@mail.tsinghua.edu.cn

[#] These authors contributed equally to this work

Conflict of interest: The authors declare no competing financial interests.

Figure legends

Figure S1. Activity-dependent repression of nrxn1α in primary cortical neuron culture. Figure 1A. RT-PCR analysis of the nrxns and other gene transcripts in primary cortical neuron cultures 24 hours after stimuli by high K^+ (51mM, 10min). Real-time qPCR analysis revealed a decrease of nrxn1α and nrxn3β mRNA after high K^+ stimulation. Figure 1B. Primary cortical neuron cultures were subjected to transient stimulation by KCl or NaCl on DIV9. The nrxn1α expression was analyzed by RT-PCR after stimulation.

Figure S2. Synthetic ZFP-based chromatin purification identified Ash1L as the transcriptional regulator of nrxn1α in mouse brain. Figure. 2A. Electrophoretic mobility shift assays was performed to test the binding capacity of the designed GST-ZFP-pnrxn1α. The PCR product of nrxn1α promoter fragment (nrx1α promoter) was incubated with GST-ZFP-pnrxn1α or GST at indicated concentrations. Lane 1, no protein; lane 2-6, 0.02, 0.04, 0.08, 0.12, 0.16 μM GST-ZFP-pnrxn1α respectively; lane 7-10, 0.04, 0.08, 0.12, 0.16 μM GST respectively. Figure 2B. Competition assays for EMSA. The DNA probes with different paired-end tags were incubated with 0.08 μM GST-ZFP-pnrxn1α. Figure 2C. The shift bands were subjected to semi-quantitative PCR to identify the ID of binding probes. Figure 2F. PCR from purified chromatins showed specific enrichment of the promoter region of nrxn1α in GST-ZFP-pnrxn1α pull-down samples. Figure 2G. Proteins associated with the nrxn1α promoter were resolved by the SDS-PAGE and silver staining. Figure 2H. ChIP analysis showed the identified proteins specific enriched at nrxn1α promoter in the hippocampus of 3-month-old C57BL/6 mice.

Figure S3. Neuronal activity recruits Ash1L and H3K36me2 to the nrxn1α promoter. Figure 3A-3B. ChIP experiments revealed the changes of histone modifications (3A) and Ash1L (3B) in the nrxn1α promoter 24 hours after high K^+ stimuli in primary neuronal cultures (E16, ICR strain).

Figure S4. Knockdown of Ash1L reduced neuronal activity-induced repression of nrxn1α. Figure 4C Reduction of protein levels 48 hours after induction in NG108-15 cells.

Figure S5. The generation of the Ash1L mutant mice. Figure 5B. A primer-pair of Ash1L-Mt-F/R was used to distinguish between the wild-type allele and the 11 deletion allele. Figure 5D. Western blot analysis of Ash1L in the motor cortex and hippocampus in 3-month-old WT mice and *Ash1L* (-/+) mice. Figure S5D. Western blot analysis of Ash1L in the thalamus and hypothalamus in 3-month-old WT mice and *Ash1L* (-/+) mice.

Figure S6. Increased nrxn1 expression and reduced H3K36me2 at the nrnx1α promoter in the hippocampus of *Ash1L* (-/+) mice. Figure 6B. Samples form the hippocampus showed no changes in histone methylation level in *Ash1L* (-/+) mice.

Figure S7. Ash1L mediated the activity-dependent repression of nrxn1α. Figure 7A. PCR genotyping of E14 embryos from *Ash1L* (-/+) intercrosses.

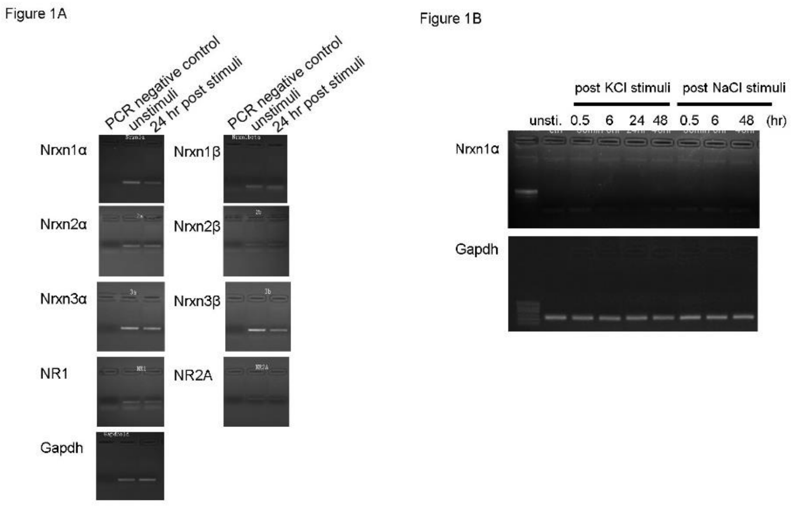

Figure S1. Full-length gels of Figure 1.

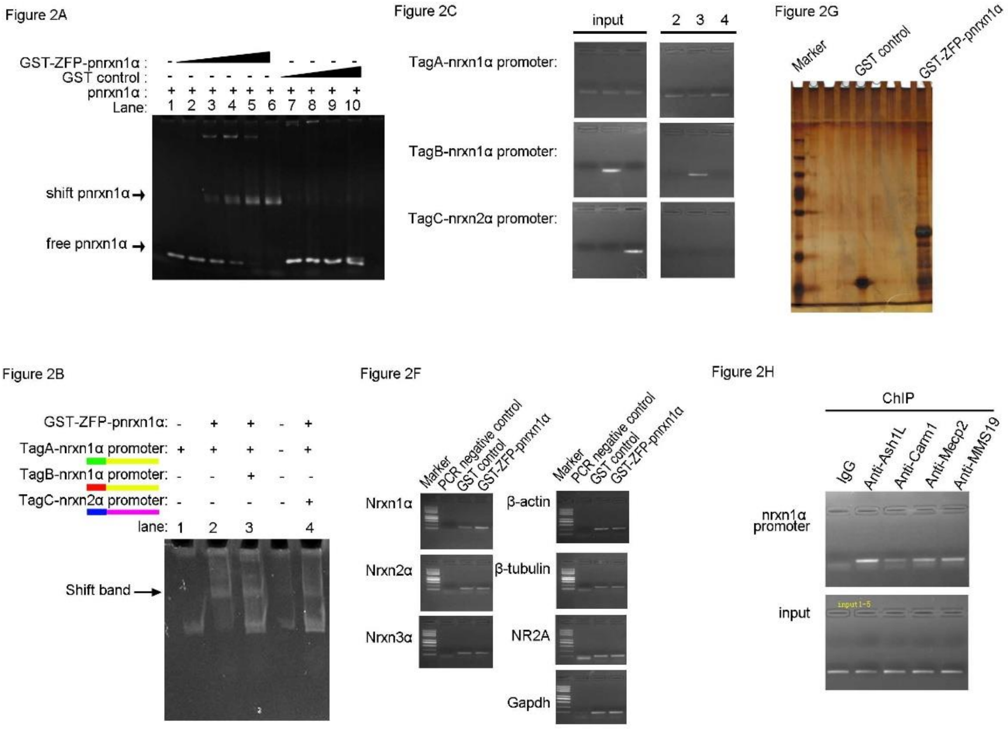

Figure S2. Full-length gels of Figure 2.

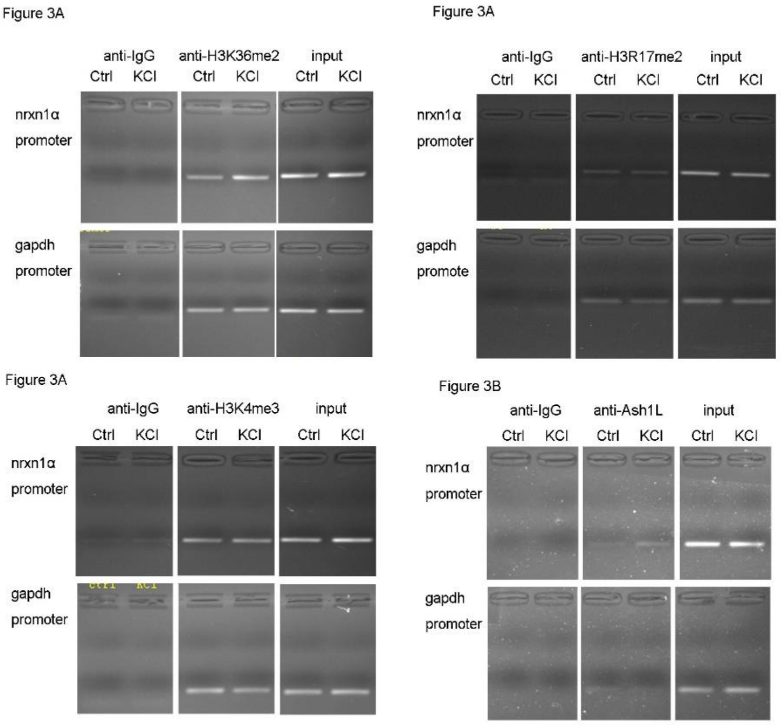

Figure S3. Full-length gels of Figure 3.

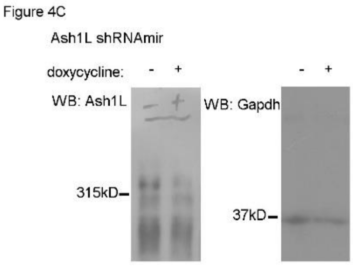

Figure S4. Full-length Western blots of Figure 4.

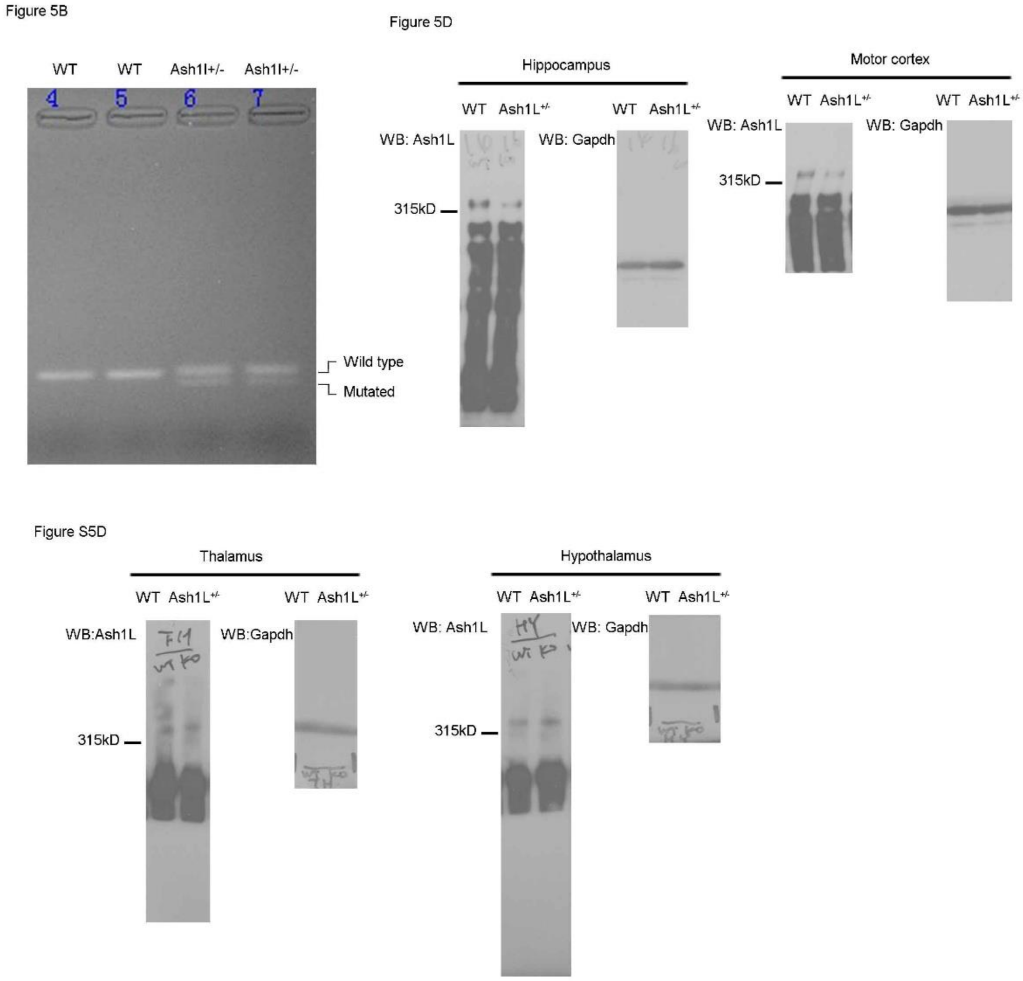

Figure S5. Full-length gel and Western blots of Figure 5.

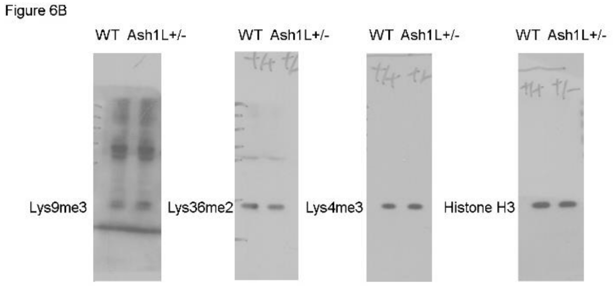

Figure S6. Full-length Western blots of Figure 6B.

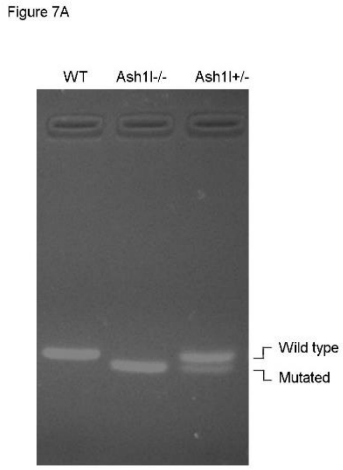

Figure S7. Full-length gel of Figure 7.
